# Accuracy and Limitations of the Growth Hormone (GH) Releasing Hormone-Arginine Retesting in Young Adults With Childhood-Onset GH Deficiency

**DOI:** 10.3389/fendo.2019.00525

**Published:** 2019-07-31

**Authors:** Giuseppa Patti, Serena Noli, Donatella Capalbo, Anna Maria Elsa Allegri, Flavia Napoli, Marco Cappa, Grazia Maria Ubertini, Annalisa Gallizia, Sara Notarnicola, Anastasia Ibba, Marco Crocco, Stefano Parodi, Mariacarolina Salerno, Sandro Loche, Maria Luisa Garré, Elena Tornari, Mohamad Maghnie, Natascia Di Iorgi

**Affiliations:** ^1^Department of Pediatrics, IRCCS Istituto Giannina Gaslini Institute, University of Genova, Genova, Italy; ^2^Department of Pediatrics, Federico II University, Naples, Italy; ^3^Department of Pediatrics, IRCCS Istituto Giannina Gaslini Institute, Genova, Italy; ^4^Unit of Endocrinology, Bambino Gesù Children's Hospital, IRCCS, Rome, Italy; ^5^SSD Endocrinologia Pediatrica, Ospedale Pediatrico Microcitemico “A. Cao,” Azienda Ospedaliera Brotzu, Cagliari, Italy; ^6^Epidemiology and Biostatistics Unit, IRCCS Istituto Giannina Gaslini, Genova, Italy; ^7^Department of Translational Medical Sciences-Pediatric Section, University of Naples Federico II, Naples, Italy; ^8^Dipartimento di Neuroncologia, IRCCS Istituto Giannina Gaslini, Genova, Italy; ^9^Health Science Department (DISSAL), University of Genova, Genova, Italy

**Keywords:** GH deficiency, transition, GHRH-arg, young adults, brain tumors

## Abstract

**Background:** Re-testing for GH secretion is needed to confirm the diagnosis of GH deficiency (GHD) after adult height achievement in childhood-onset GHD (COGHD).

**Aim:** To define the cut-off of GH peak after retesting with GH-releasing hormone plus arginine (GHRHarg) in the diagnosis of permanent GHD in COGHD of different etiology.

**Patients and methods:** Eighty-eight COGHD (median age 17.2 y), 29 idiopathic GHD (IGHD), 44 cancer survivors (TGHD) and 15 congenital GHD (CGHD) were enrolled in the study; 54 had isolated GHD (iGHD) and 34 had multiple pituitary hormone deficiencies (MPHD). All were tested with insulin tolerance test (ITT) and GHRHarg. IGHD with a GH response to ITT ≥6μg/L were considered true negatives and served as the control group, and patients with a GH response <6μg/L as true positives. Baseline IGF-I was also measured. The diagnostic accuracy of GHRHarg testing and of IGF-I SDS in patients with GHD of different etiologies was evaluated by ROC analysis.

**Results:** Forty-six subjects with a GH peak to ITT ≥6μg/L and 42 with GH peak <6 μg/L showed a GH peak after GHRHarg between 8.8–124μg/L and 0.3–26.3μg/L, respectively; 29 IGHD were true negatives, 42 were true positives and 17 with a high likelihood GHD showed a GH peak to ITT ≥6μg/L. ROC analysis based on the etiology indicated the best diagnostic accuracy for peak GH cutoffs after GHRHarg of 25.3 μg/L in CGHD, 15.7 in TGHD, and 13.8 in MPHD, and for IGF-1 SDS at −2.1 in CGHD, −1.5 in TGHD, and −1.9 in MPHD.

**Conclusions:** Our findings indicate that the best cut-off for GH peak after retesting with GHRHarg changes according to the etiology of GHD during the transition age. Based on these results the diagnostic accuracy of GHRHarg remains questionable.

## Introduction

Growth hormone deficiency (GHD) is a well-recognized clinical entity in adults. It is characterized by abnormalities in substrate metabolism, body composition, physical, and psychosocial functioning, all of which improve after GH replacement (1). Likewise, there is evidence that in young patients with persistent GHD full somatic maturation is not achieved if GH replacement therapy is discontinued after final height achievement. It is therefore recommended to evaluate the need to continuing GH replacement after completion of growth (age between 15 and 25 years) (1).

The last international consensus statement (2), recommended re-testing for GH secretion young adults with childhood-onset GHD (COGHD) and evidence of hypothalamic-pituitary disease for whom there is intention to treat. This includes patients (1) with signs and symptoms of hypothalamic-pituitary disease from endocrine, structural, and/or genetic causes; (2) who received cranial irradiation or brain tumor treatment; and (3) who presented traumatic brain injury or subarachnoid hemorrhage. The consensus recommended to continue GH replacement therapy, without the need for GH re-testing, in young adults with a transcription factor mutation, in those with more than three pituitary hormone deficits, and in those with isolated GHD associated with an identified genetic mutation.

A cutoff value of <6.0 μg/L after insulin tolerance test (ITT) was suggested to confirm the diagnosis of GHD during the transition period (2). However, since this indication was based on data obtained on a small cohort (3), further validation was recommended (2). Some later studies confirmed that this suggested cutoff was accurate to diagnose GHD in patients with high likelihood of permanent GHD, i.e., those with COGHD and structural hypothalamic-pituitary abnormalities (4, 5). Another study suggested that this value might not be reliable for the diagnosis of permanent GHD in the transition age (6). In a more recent study performed in a larger cohort of COGHD, ROC curve analysis indicated the best diagnostic accuracy for a GH peak after ITT of 5.62 μg/liter (7) confirming that the GH peak proposed by the Consensus was adequate for the definition of permanent GHD in young adults with COGHD (2). Finally, a systematic review from an Endocrine Society taskforce stated that insufficient data are available to assess the accuracy of serial GH testing in survivors of childhood cancers (8).

A number of studies have shown that patients with COGHD may have normal GH secretion when re-evaluated at the end of adult height achievement (4–7, 10). Therefore, re-testing for GH secretion is recommended to confirm the diagnosis of GHD in adolescents or young adults with COGHD. However, there is still controversy about which limit for a normal response should be considered in this age group. The ITT cut-off proposed by the 2007 Consensus (2) was first established by our group in a small cohort of 26 subjects with COGHD and high likelihood of permanent GHD compared with 39 controls (3). The GH peak of 6.1 μg/liter was the highest observed value in the patients and the lowest in the controls, with 96% sensitivity and 100% specificity (3).

ITT evaluates the integrity of the hypothalamic–pituitary axis, but it is contraindicated in patients with ischemic heart disease or seizures, and, thus, other testing modalities should be considered. Combined GH-releasing hormone plus arginine (GHRHarg) has been proposed as an alternative. We have previously shown that the cut-off limit for the normal GH response after GHRHarg was ≥19 μg/L (9), a value derived from a study performed in a large cohort of patients with normal BMI, and high likelihood of permanent GHD. Other studies, however, suggested that GHRHarg could be unreliable in the diagnosis of children and adults with COGHD (10, 11). A cut-off of <9 μg/L has been proposed for adults with childhood cancer and brain irradiation (12, 13), and a study by Darzy et al. (14) showed a high rate of false negative diagnosis when GH secretion is explored early after cranial irradiation. Since BMI has a strong negative influence on the GH response to stimulation testing (15, 16), normal limits for the GH response to GHRHarg corrected for BMI have also been proposed in adult patients (17). The American Association of Clinical Endocrinologist guidelines indicated that the BMI-based cut-off limits were <11, <8, and <4 μg/L in adult patients with BMI <25, between 25–30, and >30 kg/m^2^, respectively (17), while BMI-based normal limits in young adults who achieved final height are still lacking.

The aim of the study was to evaluate the reliability of the GHRHarg testing in the diagnosis of permanent GHD in COGHD based on (1) their GH response to ITT <6 μg/L (used as the gold standard), (2) on the underlying etiology associated with the presence of known risk factors for GHD (2), and (3) the presence of isolated GHD or multiple pituitary hormone deficiencies. To this end, we investigated in a multicenter study a large cohort of patients who underwent ITT and GHRHarg stimulation testing after achievement of adult height.

## Patients and Methods

### Subjects

This is a multicenter cross-sectional study performed in 5 Italian Pediatric Endocrine Centers: Istituto Giannina Gaslini, Genova, Federico II University, Napoli, Ospedale Pediatrico Bambino Gesù, Roma, Ospedale Pediatrico Microcitemico, Cagliari. Subjects with COGHD who required GH treatment during infancy-adolescence, aged 15–25 years and reached pubertal maturity (Tanner stage 4–5) and adult height (defined as a growth velocity below 2 cm in the previous year), were included in the study.

Subjects with COGHD for whom ITT was contraindicated (seizures or cardiac diseases) as well as patients who underwent GHRHarg testing alone or with tests other than ITT were excluded from the study. Based on the above criteria, 88 patients (39 females, 49 males) among 129 eligible subjects were enrolled in the analysis.

At the time of reassessment of GH secretory status, the patients with idiopathic GHD (IGHD) with a GH response to ITT at retesting ≥6 μg/L (2–5), normal MRI of the hypothalamic-pituitary region and no other risk factors such as cranial or craniospinal irradiation, were classified as a non-morbid control group. In the statistical analysis, these subjects were considered as true negatives. Patients with congenital abnormalities of the hypothalamic-pituitary region (CGHD) [ectopic posterior pituitary (EPP), *n* = 15], and those with CNS tumors or previous cranial or craniospinal irradiation (tumoral GHD, TGHD; *n* = 44) with peak GH to ITT <6 μg/L were considered as true positives.

The diagnosis of GHD during childhood was based on the well-recognized international criteria reported in other previous studies (4–6). Mean age at diagnosis of idiopathic GHD (non-morbid control group) was 7.9 ± 1.8 years, with a mean height SDS of −2.4 ± 1.8 years and bone age of 5.7 ± 1.0 years; mean age at diagnosis of CGHD was 4.2 ± 1.2 years, with a mean height SDS of −3.0 ± 0.6 years and bone age of 2.5 ± 0.5 years. Mean age at diagnosis of GHD in the 44 TGHD patients was 10.8 ± 2.3 years, with a mean height SDS of −1.9 ± 1.1 years and bone age of 8.3 ± 0.8 years; among the 32 irradiated patients, data on effective radiotherapy dose was available in 29 TGHD patients. Pituitary dosimetry was estimated on isodoses calculated at cranial radiotherapy (CRT) planning for 3 Dimensional conformational radiotherapy (RT-3D).

GHD diagnosis in childhood was established by a peak GH response of less than 10 μg/liter after Arginine, ITT, glucagon or clonidine tests based on the age at presentation or in the presence of contraindications. All tests were performed between 08:00 and 09:00 after overnight fasting. Arginine was administered intravenously (0.5 g/kg, max 30 g) during 30 min and blood samples for GH determination were collected at times −30, 0, 15, 30, 45, 60, 90, and 120 min. Insulin was administered intravenously (0.05–0.1 U/kg) and blood samples for GH and glucose determinations were collected at times 0, 30, 60, 90, and 120 min. A nadir glucose value during ITT below 40 mg/dL (2.2 mmol/L) was recorded in all subjects at time 30 min. The glucagon was administered im at the dose of 30mcg/kg glucagon (maximum 1 mg) and blood samples for GH, cortisol, and blood glucose were collected at time 0 and at 60, 90, 120, 150, and 180 min. Clonidine was administered orally (0.15 mg/m^2^) and blood samples for GH determination were collected at times 0, 30, 60, 90, and 120 min. Stimulation tests were performed on separate days (at least 2 days apart). The diagnosis of GHD was based on clinical characteristics and a peak GH <10 μg/L after two stimulation tests.

According to the 2007 WHO classification of tumors of the CNS (18), patients with brain tumors (*n* = 42) included embryonal tumors (*n* = 23, 19 medulloblastoma, 2 Langerhans cell histiocytosis, 2 PNET), germ cell tumors (*n* = 8, 6 germinoma), tumors of the sellar region (*n* = 4, all craniopharyngioma), ependymal tumors (*n* = 2), astrocytic tumors (*n* = 4 low grade glioma), mesenchymal tumors (*n* = 1). The hematopoietic neoplasms (*n* = 2) included one patient with acute lymphoblastic leukemia and one with acute myeloid leukemia.

Fifty-four out of 88 patients had isolated GHD (iGHD) whereas 34 had multiple pituitary hormone defects (MPHD). Among the patients with MPHD, 26 had hypothyroidism, 19 had adrenal insufficiency, 15 had hypogonadism, and 11 had diabetes insipidus. One, 2, 3, and 4 additional hormone deficits were present in 14, 9, 5, and 6 patients, respectively. All patients with MPHD were receiving conventional replacement therapy with L-thyroxine (range 30–90 mcg/m^2^), hydrocortisone (7-9 mg/m^2^/day), testosterone enanthate (250 mg/i.m./3 weeks), ethinyl estradiol (up to 30 mcg/day for 21 days/month) or transdermal 17β-estradiol patches (50 mcg/day for 21 days/month) with medroxyprogesterone acetate for females (2.5–10 mg/day for 11 days/month), and desmopressin acetate (range 30–120 mcg 3 times/day), as needed.

### Methods

The study was approved by the Ethical Committee of Istituto Giannina Gaslini and written informed consent was obtained from the patients prior to the study (code IGG MOMA 003). All patients included in the study were re-tested for GH secretion at least 1 month after GH treatment discontinuation. Demographic data (date of birth, primary diagnosis, date of primary diagnosis, date of GHD diagnosis, date of last GH treatment, date of GH re-testing) were recorded, and anthropometric measures (Height SDS, BMI and BMI SDS) were obtained.

All patients underwent ITT and GHRHarg testing on separate days as previously described (4, 5, 9–11). The tests were performed after midnight fasting and omitting their morning medications, with the exception of hydrocortisone in patients with adrenal insufficiency. IGF-I concentrations were also measured at baseline. All samples were collected, and centralized at the laboratory of Istituto Giannina Gaslini, Genova and stored at −80° until analyzed.

### Assays

Serum GH was measured by chemiluminescent immunometric assay (Immulite 2000, growth hormone; Diagnostic Products Corporation, Los Angeles, CA; international reference preparation 98/574). The inter- and intra-assay coefficients of variation were 4.2–6.6 and 2.9–4.6%, respectively, at GH concentrations of 2.6–17 μg/L. All samples from each individual subject were analyzed together at the same time. Serum IGF-I was measured by chemiluminescent immunometric assay (Immulite 2000; Diagnostic Products Corporation). The intra- and inter-assay coefficients of variation were 3.4 and 7.1%, respectively, and the sensitivity of the method was 2.6 nmol/L. After centrifugation at 4 C, plasma was separated and stored at 20 C. Serum glucose was measured automatically with a hexokinase catalyzed-glucose oxidase method.

### Statistical Analysis

Patients' characteristics were collected at the time of their recruitment. Weight, height, target height and BMI were converted in SDS based on Italian standards (19).

IGF-I-SDS was calculated using the normative data for the analytical method described by Bidlingmaier et al. (20). Statistical analyses were performed also based on the underlying etiology such as CGHD, TGHD and based on the number of pituitary defects i.e. isolated GHD or MPHD, independently of GH peak response to ITT.

Descriptive statistics were reported as frequencies and absolute numbers for qualitative variables. Quantitative variables were non-normally distributed and were expressed as median and interquartile range (IQRs) (the distance between the 25 and 75th percentile). Comparison of median values between different categories was performed using the Mann-Whitney *U* test for two-group comparisons, while the Kruskal-Wallis test was used when comparing more than two groups.

The GH peak after ITT and GHRHarg and IGF-I SDS were evaluated by standard non-parametric ROC curve analysis (21). Different groups of GHD patients were considered: (1) patients with congenital growth hormone deficiency (CGHD), (2) tumoral GHD (TGHD), (3) isolated GHD, and (4) MPHD. Sensitivity, specificity and global accuracy were evaluated at the optimal ROC analysis cut-offs, corresponding to the highest value of the Youden index (21).

ROC curves of the GH peak after GHRHarg adjusted for the potential confounding effect of BMI and BMI SDS were made and analyzed by the method proposed by Janes et al. (22), which uses the residuals from a linear regression model to remove the effect of confounders (22). A *P-*value 0.05 was considered statistically significant. All tests were two-sided. Statistical analyses were performed using Stata for Windows statistical software (Stata release 9.2; Stata Corporation, College Station, TX).

## Results

**Figure 1:**
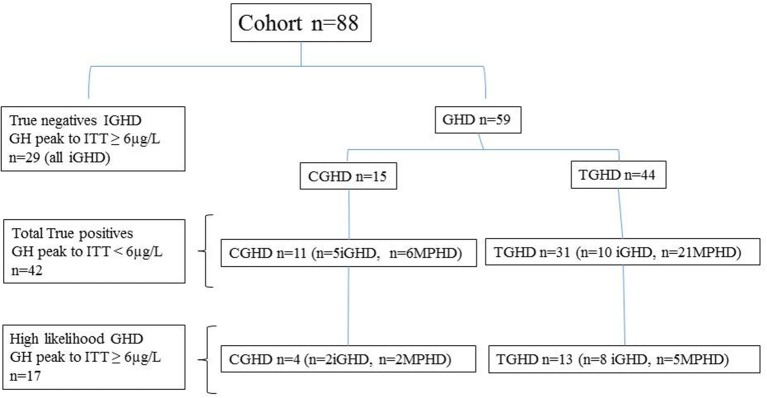
Classification of the patients based on peak GH response to ITT < or ≥6 μg/L, the underlying etiology; idiopathic GHD (IGHD), congenital GHD (CGHD), tumoral GHD (TGHD), and the presence of isolated GHD (iGHD) or multiple pituitary hormone defects (MPHD).

The characteristics of true positive patients and true negatives subjects are reported in Table 1, while those of the entire cohort based on their underlying etiologies (IGHD, CGHD, TGHD), and on the presence of isolated GHD or MPHD, independently of GH peak to ITT, are summarized in Table 2. The classification of the entire cohort is reported in Figure 1.

**Table 1:** Clinical characteristics and GH responses to ITT, GHRHarg and baseline IGF–I SDS values in the study population with GH peak to ITT < 6 μg/L (True positives).

**Study Population**		**Diagnosis**				**Pituitary Defects**		
		**True negatives**	**True positives**					
***n*** **=** **42**		**IGHD *n =* 0**	**CGHD *n =* 11**	**TGHD *n =* 31**	***p***	**iGHD *n =* 15**	**MPHD *n =* 27**	***p***
Sex (M/F)	19/23	n.e.	3/8	16/15		8/7	11/16	
Age (yrs)	17.2 (15.7 to 18.8)	n.e.	17.6 (15.4 to 20.9)	17.2 (15.7 to 18.2)	0.637	17.1 (16.0 to 20.2)	17.2 (15.6 to 18.6)	0.665
Height (SDS)	−1.1 (−2.0 to −0.5)	n.e.	−0.6 (−2.1 to −0.03)	−1.3 (−2.0 to −0.6)	0.283	−2.0 (−2.7 to −1.1)	−0.7 (−1.4 to −0.04)	0.003
Target Height (SDS)	−0.4 (−0.9 to 0.2) (*n =* 37)	n.e.	−0.5 (−0.9 to 0.4) (*n =* 8)	−0.4 (−0.9 to 0.2) (*n =* 29)	0.912	−0.4 (−0.9 to 0.03) (*n =* 14)	−0.4 (−1.0 to 0.4) (*n =* 23)	0.814
BMI (kg/m^2^)	24.9 (21.3 to 27.5)	n.e.	21.5 (19.6 to 27.9)	25.1 (22.3 to 27.4)	0.290	24.1 (20.2 to 27.5)	25.1 (22.3 to 27.9)	0.212
BMI (SDS)	1.5 (0.6 to 2.1)	n.e.	0.2 (−0.3 to 2.2)	1.5 (0.8 to 2.1)	0.161	1.4 (0.2 to 2.0)	1.5 (0.8 to 2.2)	0.294
GH peak ITT (μg/L)	1.5 (0.8 to 3.1)	n.e.	0.8 (0.1 to 2.3)	1.8 (1.0 to 3.2)	0.041	1.8 (0.8 to 3.2)	1.4 (0.6 to 2.5)	0.600
GH peak GHRHArg (μg/L)	6.8 (2.8 to 13.0)	n.e.	3.1 (2.0 to 10.1)	8.1 (4.9 to 13.4)	0.112	10.7 (2.5 to 18.9)	6.3 (2.8 to 10.9)	0.149
IGF–I (SDS)	−2.2 (−3.9 to −0.8) (*n =* 41)	n.e.	−4.3 (−5.3 to −1.3) (*n =* 11)	−2.0 (−3.6 to −0.7) (*n =* 30)	*0.094*	−1.6 (−3.1 to 0.8) (*n =* 14)	−2.8 (−4.3 to −0.7) (*n =* 27)	0.237

*IGHD, Idiopathic GHD; CGHD, congenital GHD; TGHD, tumoral GHD; iGHD, isolated; MPHD, multiple pituitary hormone deficiency*.

*Results are expressed as median (first quartile – third quartile). p = p-value (Mann-Whitney U test); n.e. = not evaluable*.

**Table 2:** Clinical characteristics and GH responses to ITT, GHRHarg and baseline IGF-I SDS values in the entire cohort based on the underlying etiology and the number of pituitary deficits.

**Study Population**			**Diagnosis**		**Pituitary Defects**	
***n =* 88**		**IGHD *n =* 29**	**CGHD *n =* 15**	**TGHD *n =* 44**	**iGHD *n =* 54**	**MPHD *n =* 34**
Sex (M/F)	49/39	17/12	6/9	26/18	32/22	17/17
Age (yrs)	17.2 (16.0 to 18.8)	17.9 (16.3 to 19.1)	17.6 (16.0 to 20.2)	16.9 (15.6 to 18.2)	17.2 (16.1 to 18.8)	17.3 (15.6 to 18.6)
Height (SDS)	−1.1 (−2.0 to −0.3)	−0.9 (−1.7 to −0.2)	−0.6 (−2.1 to −0.04)	−1.3 (−2.2 to −0.5)	−1.4 (−2.2 to −0.4)	−0.8 (−1.4 to −0.3)
Target Height (SDS)	−0.4 (−1.0 to 0.3) (*n =* 69)	−1.0 (−1.9 to 0.3) (*n =* 19)	−0.4 (−0.7 to 0.03) (*n =* 9)	−0.3 (−0.9 to 0.4) (*n =* 41)	−0.5 (−1.0 to 0.2) (*n =* 41)	−0.4 (−1.0 to 0.5) (*n =* 28)
BMI (kg/m^2^) ^c, d^	22.7 (20.1 to 25.5)	20.2 (19.2 to 23.0)	23.5 (19.8 to 27.9)	24.1 (21.0 to 26.6)	21.4 (19.3 to 24.1)	24.6 (22.1 to 26.7)
BMI (SDS)^b, c^	0.9 (−0.2 to 1.6)	−0.04 (−0.8 to 1.0)	1.3 (−0.3 to 2.2)	1.3 (0.6 to 1.9)	0.4 (−0.6 to 1.3)	1.4 (0.6 to 2.1)
GH peak ITT (μg/L)^a, b^	6.5 (1.6 to 14.4)	17.1 (11.4 to 22.4)	1.4 (0.1 to 7.9)	3.1 (1.4 to 6.8)	10.5 (4.6 to 17.3)	2.1 (1.0 to 4.9)
GH peak GHRHArg (μg/L)^a, b^)	17.4 (6.9 to 40.0)	40.0 (31.4 to 40.0)	6.7 (2.5 to 19.6)	10.8 (5.8 to 19.7)	30.0 (16.6 to 40.0)	7.5 (3.6 to 13.0)
IGF–I (SDS)^b, c^	−1.1 (−2.4 to −0.2)	−0.5 (−1.1 to 0.4) (*n =* 27)	−2.4 (−4.6 to −1.0) (*n =* 15)	−1.6 (−3.1 to −0.5) (*n =* 43)	−0.7 (−1.7 to 0.1)	−2.2 (−3.8 to −0.7)

*IGHD, Idiopathic GHD; CGHD, congenital GHD; TGHD, tumoral GHD, iGHD, isolated; MPHD, multiple pituitary hormone deficiency*.

*Results are expressed as median (first quartile – third quartile)*.

*P-value by Kruskal-Wallis (IGHD vs. TGHD vs. CGHD) = a < 0.001; c < 0.01; by Mann-Whitney (iGHD vs. MPHD) b < 0.001; d < 0.01*.

All subjects reached a nadir blood glucose level during ITT <2.2 mmol/liter (40 mg/dl). Based on the GH response to ITT < or ≥6 μg/L, 29 patients with IGHD showed a GH peak ≥6 μg/L (true negatives) and 11 CGHD and 31 TGHD had a GH peak <6 μg/L (true positives). In particular, true positives CGHD showed a GH peak to ITT significantly lower compared to true positives TGHD (Table 1). Four out of 15 CGHD (*n* = 2 isolated GHD; *n* = 2 MPHD) and 13 out of 44 TGHD (*n* = 8 isolated GHD, *n* = 5 MPHD) showed a GH response to ITT ≥6 μg/L (Figure 1) despite the presence of known risk factors for high likelihood GHD as defined by the consensus (1), i.e. severe GHD in childhood with or without two or three additional hormone deficits, possibly due to a genetic abnormality, structural hypothalamic-pituitary abnormalities, central nervous system tumors or after high-dose cranial irradiation.

Median GH peak after ITT was significantly lower in CGHD and TGHD patients than in IGHD subjects (Table 2).

### GHRHarg Testing

**Figure 2:**
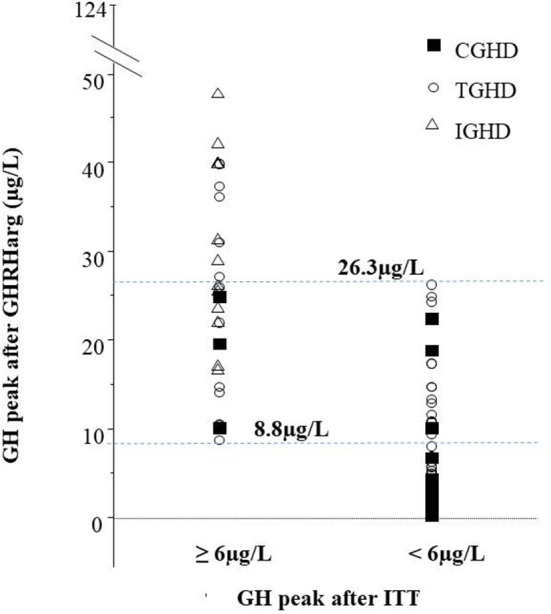
GH peak to GHRHarg in the cohort based on the GH response to ITT ≥6 μg/L or <6 μg/L. The peak GH to GHRHarg values between 8.8 μg/L (lowest peak in subjects with GH ≥ 6 μg/L) and 26.3 μg/L (highest peak in patients with GH <6 μg/L) are represented; 29 subjects with idiopathic GHD (open triangles), 15 with congenital GHD (closed squares), and 44 Tumoral GHD (open circles).

Figure 2 shows the distribution of GH peak to GHRHarg based on the ITT response < or ≥6 μg/L. Regarless of the underlying etiology there was a wide overlap of GH values between 8.8 and 26.3 μg/L. Forty-six subjects [17 of whom with a high likelihood GHD such as CGHD (*n* = 4), and TGHD (*n* = 13)], showed a GH peak to ITT ≥6 μg/L and 42 <6 μg/L (true positive) with a peak GH after GHRHarg ranging between 8.7–124 μg/L and 0.3–26.3 μg/L, respectively (Figure 2). In the true positive GHD subjects GH peak responses to GHRHarg ranged between 0.3 and 26.3 μg/L; in particular, GH peak was lower in CGHD (median 3.1, range 0.3–22.5 μg/L) compared to TGHD (median 8.1, range 0.6–26.3 μg/L) (Table 1).

The median GH response to GHRHarg in the entire cohort based on their underlying etiology and the number of pituitary defects is reported in Table 2 In particular, the GH response was significantly lower in CGHD and TGHD patients than in IGHD subjects and in MPHD patients compared to isolated GHD.

**Figure 3:**
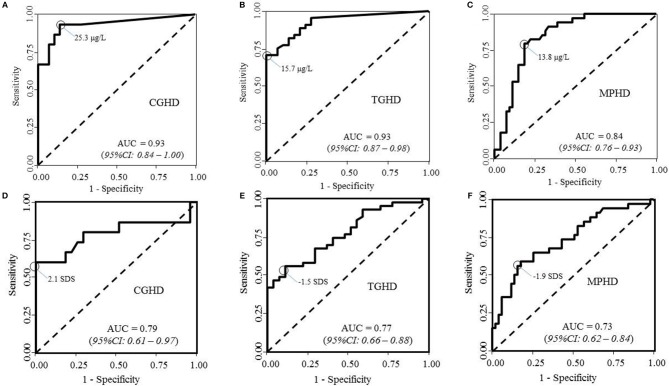
ROC curve analyses of peak GH after GHRHarg and IGF-I SDS in **(A,D)** patients with congenital growth hormone deficiency (CGHD, **A,D**), tumoral GHD **(B,E)**, and **(C,F)** multiple pituitary hormone deficiency (MPHD).

The results of ROC curve analysis of GHRHarg in patients with CGHD (panel a), TGHD (panel b), and MPHD (panel c) are reported in Figure 3. The highest proportion of correctly classified CGHD patients (88.6%**)** was obtained for a GH peak cutoff of 25.3 μg/liter (95% CI, 13.4–27.0; sensitivity, 93.3%, 95% CI: 82.7–100%; specificity, 86.2%, 95% CI: 75.7–96.8%; AUC 0.93; 95% CI, 0.84–1.00). It was 82.1% in TGHD patients for a GH peak of 15.7 μg/liter (95% CI, 15.6–38.7; sensitivity, 70.5%, 95% CI: 59.2–81.8%; specificity, 100.0%; AUC 0.93; 95% CI, 0.87– 0.98), and of 80.7% in MPHD patients for a GH peak of 13.8 μg/liter (95% CI, 12.5–25.3; sensitivity, 79.4%, 95%CI: 68.0–90.8%; specificity, 80.5%, 95% CI: 72.8–90.2%; AUC 0.84; 95% CI, 0.76–0.92).

### BMI and GH Response to GHRHarg

There was a significant inverse correlation between BMI SDS and GH peak responses to GHRHarg (*r* = −0.43, *p* < 0.0001) when considering the entire cohort; a similar correlation (*r* = −0.43; *p* = 0.003) was found in TGHD patients. Based on these findings ROC curves were adjusted for either BMI or BMI SDS, showing that obesity could be a confounder (=lower peak) in interpreting the GH response in obese patients with MPHD (AUC 0.78 vs. 0.84; 95% CI, 0.67–0.89, and AUC 0.79 vs. 0.84; 95% CI, 0.68–0.89, respectively) (data not shown). A significant inverse correlation between BMI SDS and GH peak response was also found after ITT (*r* = −0.43, *p* < 0.0001) in the whole cohort and in TGHD (*r* = −0.33; *p* = 0.03).

### IGF-I SDS

True positives CGHD had lower IGF-1 SDS (median −4.3, range −8.1 to −1.1) compared to true positives TGHD (median −2.0, range −7.1 to 1.8, *p* = 0.094) (Table 1). The median IGF-I SDS in the entire cohort based on their underlying etiology and the number of pituitary defects is reported in Table 2. Median IGF-I SDS was significantly lower in CGHD and TGHD patients than in IGHD subjects (−0.5; IQR, −1.1–0.4) (*p* < 0.0001).

Results of ROC analysis in patients with CGHD (Figure 3D), TGHD (Figure 3E), and MPHD (Figure 3F) are reported in Figure 3. IGF-I SDS showed the best diagnostic accuracy for CGHD (85.7% correctly classified subjects) with an optimal IGF-I SDS cutoff of−2.1 SDS (sensitivity, 60.0%, 95%CI: 39.3–80.7%; specificity 100%; AUC 0.79; 95% CI, 0.61–0.97). The best diagnostic accuracy was 68.6% in TGHD patients for an IGF-I SDS of −1.5 SDS (sensitivity, 55.8%, 95%CI: 43.4–68.2%; specificity 82.4%, 95%CI: 73.7–91.2%; AUC 0.73; 95% CI, 0.62–0.84); and specificity 88.9%, 95%CI: 80.0– 98.8%; AUC 0.77; 95% CI, 0.66–0.88), 72.9% in MPHD patients with an IGF-I SDS of −1.9 SDS (sensitivity, 58.8%, 95%CI: 45.0–72.6%).

### Cranial Radiotherapy and GH Responses

**Figure 4:**
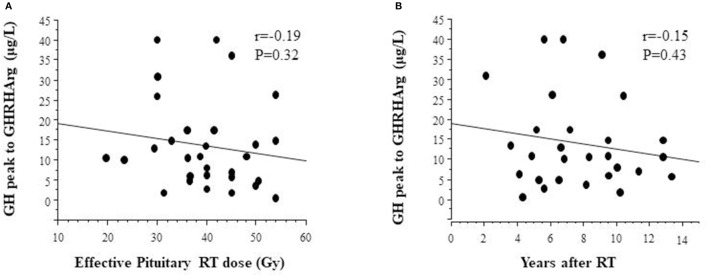
Relationship between GH peak responses to GHRHarg and **(A)** effective radiotherapy dose, and **(B)** distance (years) after radiotherapy.

Anthropometric measures and GH responses to GHRHarg in 29 TGHD patients (*n* = 12 females) with effective pituitary radiotherapy dose are reported in Table 3; tumor diagnosis were medulloblastomas (*n* = 14), geminomas (*n* = 6), craniopharyngioma (*n* = 2), low grade gliomas (*n* = 3), PNET (*n* = 2), other (*n* = 2). Effective pituitary RT dose was inversely but not significantly related to the GH response after GHRHarg (Figure 4), with a similar negative trend between the GH response and years after RT (Figure 4).

**Table 3:** Clinical characteristics and GH responses to GHRHarg in 29 patients with effective RT pituitary dose.

Age at re-assessment, years	17.2 (15.9 to 18.7)
Age at off therapy, years	9.8 (6.8 to 11.9)
GHRHArg (GH peak), μg/L	17.4 (6.9 to 40.0)
IGF-I SDS	−1.1 (−2.5 to −0.2)
Height, SDS	−1.1 (−2.0 to −0.3)
BMI SDS	0.9 (−0.2 to 1.6)
Effective pituitary RT dose, Gy	40.0 (35.3 to 45.8)
Years after RT, years	6.8 (5.3 to 9.7)

## Discussion

The GHRHarg test is one of the most powerful GH stimulation tests (23–25) and, at odds with classical provocative testing, shows less intra-individual variability (25). This test has been proposed as a reliable alternative to the classical provocative tests for the diagnosis of GHD (26), although robust normative data for late adolescence and young adulthood have not been established. Previous studies have shown that testing with GHRHarg distinguishes normal subjects from those with MPHD and that it is as sensitive as ITT for the diagnosis of GHD in adults and older adolescents (25). Indeed, our group showed that some children and young adults with congenital GHD or acquired GHD may have a “normal” GH response to GHRHarg (10, 11) suggesting that GHRHarg testing may fail to recognize all patients with permanent GHD.

The present study shows that the GH response to GHRHarg in adolescents and young adults with congenital or tumoral COGHD does not always confirm the results of ITT. In particular, the GHRHarg-induced GH secretion showed marked differences both in true positives patients and in those with different underlying etiologies with a high likelihood of permanent GHD. These findings strongly suggest that the same GH cut-off value after GHRHarg is not appropriate for the diagnosis of permanent GHD after adult height achievement in all patients with COGHD. By adopting a GH value <8.8 μg/L after GHRHarg, 24 (57%) among our true positives GHD patients would have been correctly identified, while 3 patients (27%) with CGHD and 15 (48.4%) with TGHD would have been misdiagnosed. Furthermore, our current finding in 4 CGHD or TGHD patients (10%) of a GH peak >19.0 μg/L (data not shown), are partially in agreement with those of our previous study supporting that a cut-off limit of 19.0 μg/L after GHRHarg is a reliable diagnostic test in patients with COGHD (9). These discrepancies may be due to the better definition of the subjects' characteristics of the present study which includes congenital GHD, as well as to the inclusion of a large number of irradiated cancer survivors. In addition, we are aware that GH measurement is strongly influenced by the type of assay (27). In this regard, it should be pointed out that in this study we used a monoclonal chemiluminescent immunometric assay with reference preparation 98/574 as recommended by the GRS consensus (2), and that the assay was centralized, while an immunoradiometric method was used in the previous study (9). The importance of the GH assay in the definition of normal limits has been recently confirmed in a study showing that a GH value <15.9 μg/liter after GHRHarg obtained with a conversion factor of an in-house RIA assay (28) was accurate for the definition of GHD at the time of retesting in adolescents previously treated with GHD. Furthermore, there was a significant inverse correlation between BMI SDS and peak GH responses to GHRHarg in the entire cohort as well as in MPHD and cancer survivor patients suggesting that BMI may be among the factors that influence the variability of the GH response also to stimulation with GHRHarg (15, 16). In the study of Dreismann et al. (28) patients with BMI between −1and 0 SDS showed higher GH peaks compared to those with BMI >1 SDS. On the other hand, the suggested cut-off peak after GHRHarg <19 μg/liter for the diagnosis of permanent GHD of our previous study (9), was established in lean subjects (BMI <25 kg/m^2^). Taken together these findings indicate the need of establishing cut-off limits appropriate for overweight and obese patients in/during the transition phase.

The recent Endocrine Society Clinical Practice Guideline addresses the problem of abnormalities of hypothalamic–pituitary functioning and growth disorders frequently observed in childhood cancer survivors (29). Testing with ITT, GHRH (with or without arginine), and glucagon has been recommended, in this order, for the diagnosis of GHD in adult survivors of childhood cancer (29), while no recommendations were provided for the diagnosis of GHD after adult height achievement in COGHD. In addition, a systematic review on the diagnosis of GHD as a late effect of radiotherapy in survivors of childhood cancer raises the question on how to interpret the GH response after GHRHarg in patients with primary hypothalamic dysfunction (8) with a recommendation against the use of GHRH alone or in combination with arginine after hypothalamic–pituitary axis radiation. Our study, the largest reported so far in cancer survivors retested after adult height achievement (8), confirms that patients with GHD and brain tumors have significantly lower responses of GH after GHRHarg, although some of them perform well above the recommended cut-offs (9–11, 15–17).

ROC analysis of IGF-I showed the best diagnostic accuracy for an IGF-I cut-off of ~2 SDS in congenital GHD and in patients with MPHD and of−1.45 SDS in tumoral GHD with a sensitivity, and specificity ranging from 46.6 to 100%. This suggests that IGF-I SDS cut-offs may differ based on the underlying conditions and the severity of GHD and that IGF-I SDS performs poorly in cancer survivors. These findings support the recommendations against relying the diagnosis of GHD solely on serum IGF-I levels in patients exposed to hypothalamic–pituitary axis radiotherapy (29).

Our results are representative of the impact of effective radiation dose and length of follow-up (time elapsed since irradiation) on the pattern of peak GH responses to GHRHarg showing that effective pituitary RT dose was inversely although not significantly related to GH response after GHRHarg. The non-significant negative trend to GHRHarg and the time interval after irradiation, indicates that somatotroph dysfunction is time-dependent and progressive. Indeed, the absence of a significant reduction of the GH response over time in our study compared to that by Darzy et al. (14) appear to be very likely due to the longer time of follow-up as well as to the higher biological effective dose of RT used in their cohort.

Results of the present investigation should be evaluated at the light of some inevitable limits. First, the variability of the cut-offs obtained by ROC analysis was quite high, as pointed out by the large confidence intervals of their estimates. Second, the selection of the optimal cut-off makes results comparable to those of previous investigations, but does not take into account the potentially different costs of false positive and false negative errors. Third, the accuracy measured at an optimal cut-off could be overestimated. Further investigations on large independent cohorts should be carried out to confirm our results.

In conclusion, in spite of the above cited limits, our findings indicate that ITT testing in young adults with childhood onset GHD is reliable in different GHD conditions, whereas the diagnostic accuracy of GHRHarg remains questionable in several patients. The different GH cut-offs obtained by ROC analysis in congenital GHD, isolated GHD, MPHD and in cancer survivors make testing with GHRHarg poorly useful in clinical practice, and suggest the need for establishing normal GH peak values during the transition age for every underlying condition. The progressive time-dependent reduction of the GH response to GHRHarg in cancer survivors makes the interpretation of the GH secretory status a real challenge.

## Data Availability

All datasets generated for this study are included in the manuscript/supplementary files.

## Ethics Statement

The studies involving human participants were reviewed and approved by Ethical Committee of Istituto Giannina Gaslini, study (code IGG MOMA 003). Written informed consent to participate in this study was provided by the participants' legal guardian/next of kin.

## Author Contributions

GP and SNol and have contributed equally in following the patients, collecting the data, drafting and revising the manuscript. DC, MS, MCa, GU, SL, and AI have participated to the multicenter study by enrolling and taking care of their patients as well as by revising the manuscript. AA and FN take care of the patients and revised the manuscript. AG, SNot, and MCr helped in the evaluation and the follow-up of the patients. SP performed statistical analysis. MG takes care of the neuro-oncology patients. ET takes care of the neuro-oncology patients during radiotherapy. MM designed the study and actively participated to drafting and revision of the manuscript. ND designed the study, supervised the patients and actively participated in data analysis, drafting and revision of the manuscript. All authors approved the final manuscript as submitted and agree to be accountable for all aspects of the work.

### Conflict of Interest Statement

The authors declare that the research was conducted in the absence of any commercial or financial relationships that could be construed as a potential conflict of interest.
